# Impact of Groundwater Table and Plateau Zokors (*Myospalax baileyi*) on Ecosystem Respiration in the Zoige Peatlands of China

**DOI:** 10.1371/journal.pone.0115542

**Published:** 2014-12-26

**Authors:** Yan Zhou, Nana Li, John Grace, Meng Yang, Cai Lu, Xuemeng Geng, Guangchun Lei, Wei Zhu, Yongfeng Deng

**Affiliations:** 1 School of Nature Conservation, Beijing Forestry University, Beijing, China; 2 School of Geosciences, University of Edinburgh, Edinburgh, United Kingdom; 3 Management Bureau of Zoige National Nature Reserve, Sichuan, China; Swiss Federal Research Institute WSL, Switzerland

## Abstract

Peatlands contain large amount of carbon stock that is vulnerable to release into the atmosphere. Mostly because of human impact, the peatlands at Zoige Wetlands face severe degradation, and the groundwater table is now lower than before, which has increased the population of the plateau zokor, a burrowing rodent. However, the impact of these changes on ecosystem carbon flows has not been studied. To investigate how the plateau zokor and the groundwater level alter the ecosystem respiration of the Zoige peatlands, we sampled the CO_2_ flux of hummocks shaped by the zokors and compared it with the CO_2_ flux of undisturbed sites with different groundwater table levels. The soil organic carbon (SOC), soil water content (SWC) and soil temperature at 5 cm (T_5_) were measured. SOC showed no significant difference among the four sampling sites and did not correlate with the CO_2_ flux, while SWC was found to partly determine the CO_2_ flux. A linear equation could adequately describe the relationship between the natural logarithm of the ecosystem respiration and the soil temperature. It is demonstrated that descending groundwater table might accelerate ecosystem respiration and the CO_2_ flux from hummocks was higher than the CO_2_ flux from the control site in the non-growing season. With rising temperature, the CO_2_ flux from the control site accelerated faster than that from the hummocks. Our results show that ecosystem respiration was significantly lower from hummocks than at the control site in the growing season. The results on the impact of zokors on greenhouse gas emissions presented in this paper provide a useful reference to help properly manage not only this, but other litter-burrowing mammals at peatland sites.

## Introduction

The northern peatlands are thought to be the largest terrestrial carbon stock, containing 270–547 Pg C that has accumulated over approximately 10,000 years as a result of photosynthetic activity [1]–[3]. However, as global temperatures increase, decomposition of the peat is expected to increase, turning the present-day weak carbon sink into a carbon source, giving rise to an increase in the atmospheric carbon dioxide (CO_2_) concentration, and therefore accelerating global warming through a positive feedback process [4]. The global annual respiratory CO_2_ flux from soil is thought to be massive – somewhere in the region of 70 Pg C [5]. For peatland, the C balance shows an interannual variability from a weak C source to a strong C sink [6]. Moreover, the loss of carbon is exacerbated by other human impacts, including grazing, peat harvesting, acid deposition, and cultivation [7], [8].

Below-ground and above-ground respiration are influenced by various biotic and abiotic factors, such as vegetation activity [9], [10], and the quantity and quality of soil organic carbon (SOC) [11]. Human activities in peatlands usually affect soil respiration by changing the biotic or abiotic conditions of the soil [12]. A high water table might sustain the carbon stock of peatlands by preventing aerobic respiration, whilst drainage might greatly increase the CO_2_ emissions from peatland to the atmosphere, and restoration take opposite effect [13]. To complete our understanding of the greenhouse gas (GHG) emissions of peatlands, and to provide data for model simulations, it is important to collect information on ecosystem and soil respiration in peatlands where the groundwater table fluctuates widely. Such information will help to reveal the impact of drainage and restoration on the CO_2_ emissions of peatland, especially for those regions with the largest levels of C storage, like many of the high–elevation landscapes of China.

One factor that has to date received only minor attention is the impact of burrowing animals on CO_2_ emissions. Previous studies have found that animals influence GHG emissions in agricultural systems, for example through the manure produced by cattle increasing the emissions of CO_2_ [14], CH_4_ [15], [16] and N_2_O [17]. However, there is much less information on the impact of wild animals on ecosystem respiration in natural and semi-natural ecosystems.

The Zoige Marsh is a vast region of peat in the Qinghai–Tibet Plateau, utilized mostly for high-intensity grazing of yaks, horses and sheep. The highly specialized subterranean herbivore, the plateau zokor (*Myospalax baileyi*), is the only subterranean rodent species of the Qinghai–Tibet Plateau [18] and is distributed over most of the Plateau's prairies and meadows [19]. Zokors are medium-sized rodents (approximately 20 cm long, excluding the tail) that use their powerful front claws for digging and feeding on plant matter, including tubers and seeds.

The burrowing of zokors produces hummocks on the prairie and meadow, changing the landscape and biomass of the pasture, and also influencing the ecosystem functions of the pasture where livestock graze [20]. Although the plateau zokor is considered to be a harmful mammal for its burrowing and foraging, previous work has revealed that zokors are important for maintaining or restoring native plant communities by improving the soil quality [21]. However, how zokors shape the ecosystem by burrowing is still unknown, especially with respect to the influence of their activities on GHG emissions.

The main objective of this study was to evaluate the ecosystem respiration along a gradient of soil water and to clarify the impact of hummock formation by zokors on ecosystem respiration.

## Material and Methods

This study was authorized by the Management Bureau of Zoige National Nature Reserve. The field study did not involve endangered or protected species, and no specific permissions were required for the study.

### Site description and site selection

**Figure 1:**
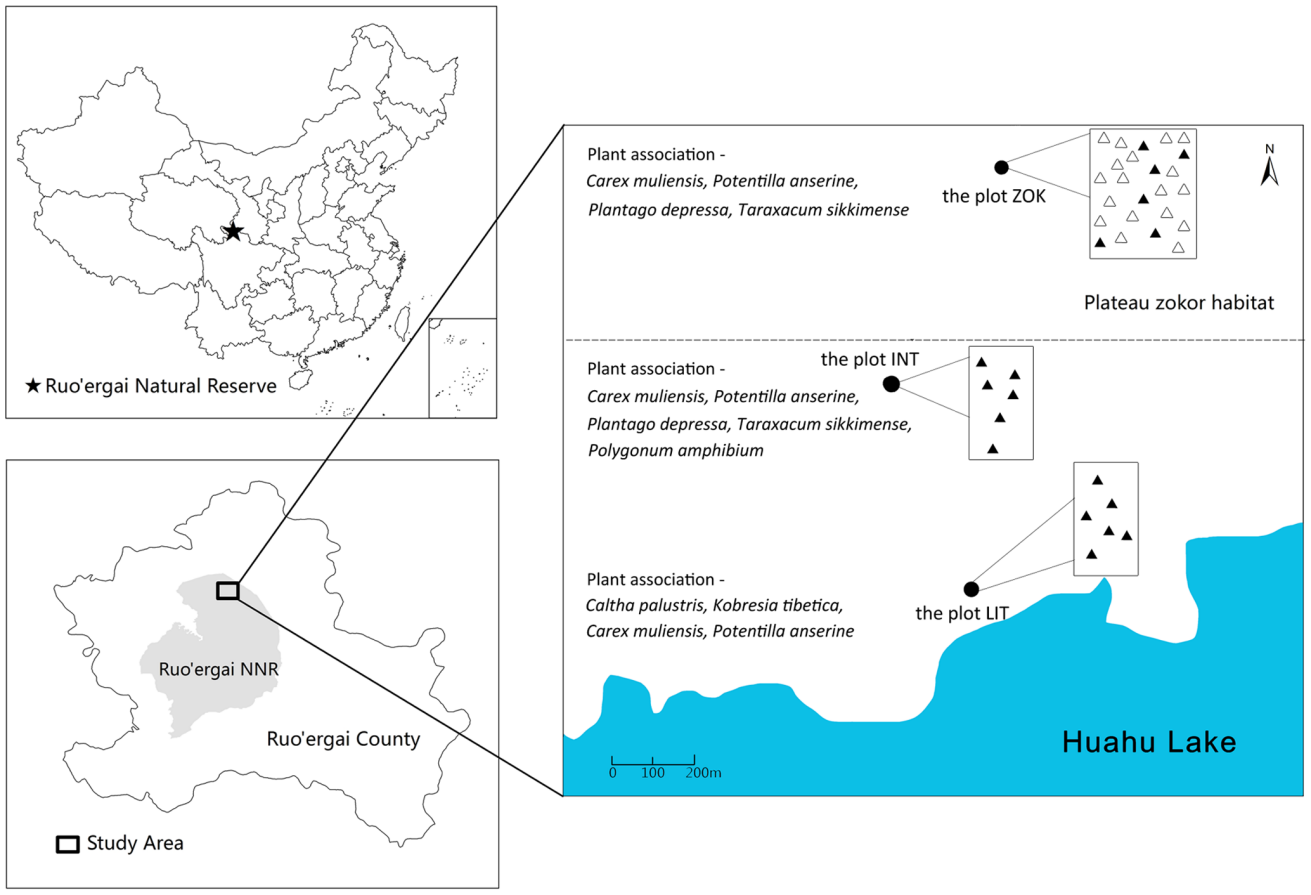
Distribution of the study sites in the Zoige peatland. The three study plots (LIT, INT and ZOK) are shown by black dots. The distance from plot ZOK (33.929°N, 102.820°E; 3439m a.s.l.) to plot INT (33.923°N, 102.817°E; 3437m a.s.l.) is approximately 700 m, and the distance from plot INT to plot LIT (33.918°N, 102.818°E; 3435m a.s.l.) is approximately 600 m. Six permanent samples were taken each time, and are marked by solid triangle at each plot, and named as LIT, INT and CON, whereas for sample HUM, six samples were taken each time, and then, move to another location to take second, and third sets of samples, which are marked by open triangle at plot ZOK. The image at left bottom shows the border line of the Zoige County and the Zoige National Nature Reserve. The blue area in the right image represents Huahu Lake, whilst plateau zokor aggregates in the area above the dot line.

The study area was close to Huahu Lake in Zoige National Nature Reserve (33.5°N, 102.5°E; 3430 m a.s.l.), approximately 40 km north of Ruoergai County town, Sichuan Province. Zoige Wetland is located in the eastern Qinghai–Tibet Plateau, southwestern China (Fig. 1). It experiences typical cold climatic conditions of the Plateau. Annual precipitation at Zoige is 650 mm and the annual average temperature is 1.7°C. There is only a three-month growing season from June to August, when most of the precipitation occurs [22].

**Figure 2:**
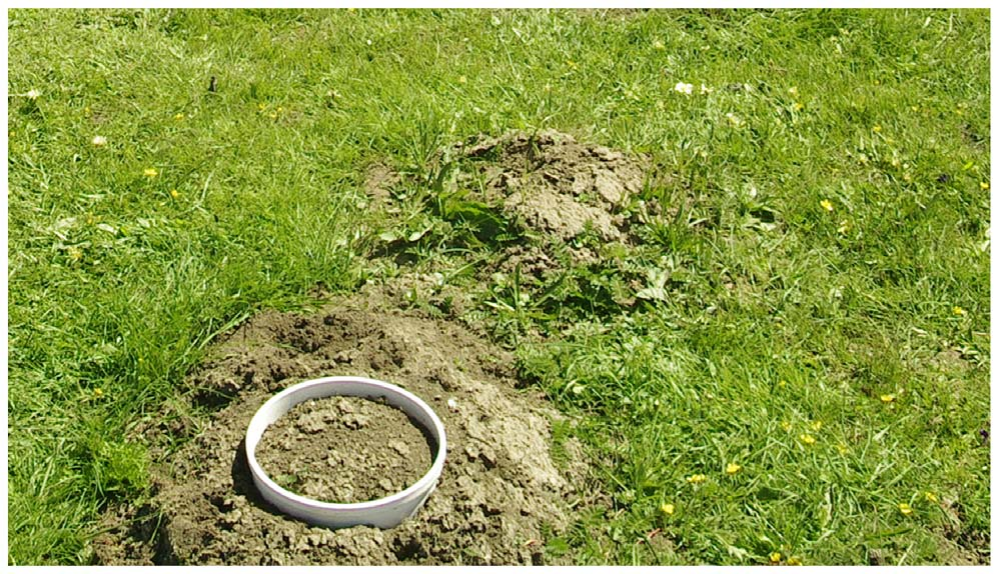
PVC tube place over the plateau zokor hummocks in the Zoige peatlands.

The groundwater table descended with the increase of the distance to Huahu Lake. Plateau zokors only inhabit in the area with low groundwater table to reduce the risks of being inundated. According to the groundwater table and distribution of plateau zokors, three plots were selected for respiration studies, representing zones with different groundwater tables (Fig. 1). These were: plot LIT, which represented the littoral zone of Huahu Lake with 20 cm groundwater table; plot ZOK is located the furthest to Huahu Lake and had the lowest groundwater table (350 cm below ground) among the three plots; and plot INT, with the groundwater table level at intermediate level (200 cm below ground), is located between plot LIT and ZOK. Plateau zokors were found in plot ZOK and therefore offers opportunity to study the effect of groundwater table, as well as the plateau zokor on the emission of CO_2_. At the plot ZOK, two sets of samples were taken, one is to measure zokor respiration (which is called HUM) was placed over the bald hummocks that are formed by plateau zokors (Fig. 2), the other set of samples is to measure the ecosystem respiration similar to plot LIT and INT, as the control samples for HUM. The predominant plant species of the plot LIT are *Caltha palustris* L., *Kobresia tibetica* Maxim, *Carex muliensis* Hand. -Mazz. and *Potentilla anserine* L., while the predominant plant species of the plot INT and ZOK are *Carex muliensis* Hand. -Mazz., *Potentilla anserine* L., *Plantago depressa* Willd. and *Taraxacum sikkimense* Hand. -Mazz. *Polygonum amphibium* L. is wildly distributed in plot INT.

### Ecosystem respiration and SOC measurement

Ecosystem respiration was measured on sunny days during June, July and October 2012 using a Li-8100 soil CO_2_ flux system (LI-COR Inc., Lincoln, NE, USA). Six replicates of tubal PVC collar (diameter: 20 cm; height: 15 cm) were placed randomly at plot LIT and INT, as well as the zokor control sample sets CON at plot ZOK, with the collars installed 2–3 cm above the ground and the distance between any two collars being no greater than 50 m. Throughout the whole sampling period, the collars remained at the same location. To ensure the measure of plateau zokor respiration, six collars were random placed on the fresh hummock (which had no plants growing on it) each month (June, July, and October), and therefore, the sampling locations for HUM were 18, compared to 6 for CON, LIT and INT. Temperature at 5 cm (T_5_) below the ground was measured adjacent to each collar with a portable temperature probe provided with the Li-8100 system. Soil water content (SWC) at the depth of 5 cm was also measured with a portable probe provided with the Li-8100 system, close to each sampling location.

Soil samples at depths of 0–10, 10–20 and 20–30 cm were collected next to each collar at sites LIT, INT and CON in July 2012. At site HUM, the collars were removed after the measurement of respiration. Soil samples from the three layers were collected at the place where the collar was installed to ensure the soil samples reflected the conditions of the zokor hummock. SOC (g/kg) was measured by the potassium-dichromate oxidation procedure after H_2_SO_4_–HClO_4_ digestion [23].

Temperature coefficient (Q_10_) was used to measure the rate of change of ecosystem respiration as a consequence of increasing the temperature by 10°C. The Q_10_ is calculated as 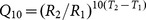, where R is ecosystem respiration (units: umol*m^−2^s^−1^) and T is the temperature in Celsius degree.

### Statistical analysis

All statistical analyses were conducted with SPSS version 17.0 [24]. Data failed the test for normality and therefore non-parametric statistics were used. The Mann–Whitney U Test was employed to compare the means of respiration at different sites and in different sampling periods. The Kruskal Wallis Test, post-hoc Dunn's multiple comparison test were applied to check SOC and the diurnal change of the CO_2_ flux. The statistical relationships between the natural logarithm of ecosystem respiration and T_5_, SWC and SOC were examined by regression analyses using a linear equation.

## Results

### SOC variability

**Figure 3:**
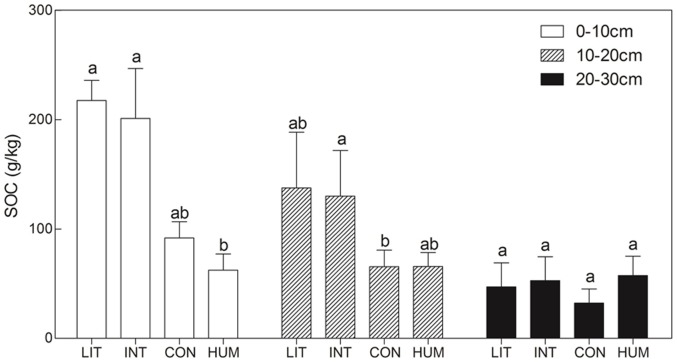
SOC (mean ± SE) compared among the sites at three depth layers. Comparison of SOC in the 0–10, 10–20 and 20–30 cm layers among the four sites. Within the same layer group, the SOC values with the same lowercase letter do not differ significantly (*P*>0.05) based on the Dunn's multiple comparison test.

SOC at all sites varied around 40–230 g/kg in growing season, and then changed to 30–90 g/kg in October. As Fig. 3 shows, the SOC significantly differed among the four site at the 0–10 cm and 10–20 cm depth soil layer (Kruskal Wallis Test, *P*<0.001), whereas no significant difference was found among the four sites at the 20–30 cm depth soil layer (Kruskal Wallis Test, *P* = 0.088). The SOC tended to be doubled at sites LIT and INT than at site CON at all soil layers (the depths of 0–10, 10–20, and 20–30 cm), but the variation was not statistically significant (Dunn's multiple comparison test, *P*>0.05), except SOC of site INT, which was significant higher than that of site CON at the depth of 10–20 cm (Dunn's multiple comparison test, *P*<0.05). At the 0–10 cm depth soil layer, SOC at site CON was higher than that at site HUM, but was still not significantly differnent (Dunn's multiple comparison test, *P*>0.05).

### Ecosystem respiration

**Figure 4:**
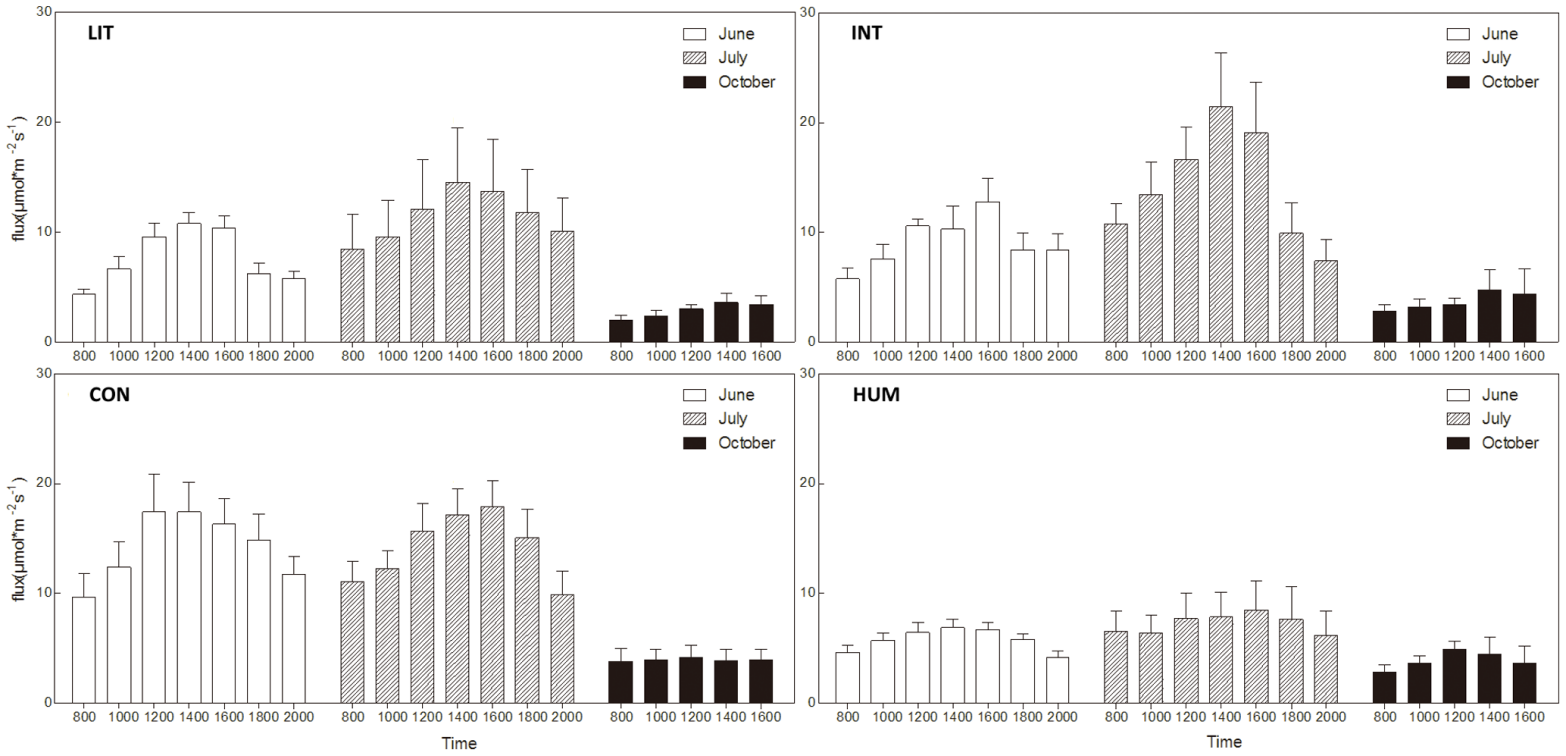
Diurnal CO_2_ flux from the four sites in 2012. The diurnal dynamics of CO_2_ flux from the four sites were collected in June, July and October 2012.

CO_2_ flux data at all sites varied around 4–25 µmol*m^−2^s^−1^ in the growing season, while the range of CO_2_ flux in October was 2–8 µmol*m^−2^s^−1^. As Fig. 4 and Table 1 show, sites CON and INT released significantly more CO_2_ than site LIT in each sampling month. In July and October, CO_2_ flux from site CON was similar to that from site INT, while the CO_2_ flux from site CON higher than that from site INT in June.

**Table 1:** The *P* values of the Mann–Whitney U Test of CO_2_ flux among the four sites.

		Sites	
Month	Sites	INT	CON
	LIT	0.023	<0.001
June	INT		<0.001
	HUM		<0.001
	LIT	0.024	0.001
July	INT		0.591
	HUM		<0.001
	LIT	0.011	<0.001
October	INT		0.181
	HUM		0.679

In the growing season of 2012, sites CON and HUM, located close to each other in the zone with the lowest groundwater table, showed differences in their rates of CO_2_ emission. Site HUM emitted significantly less CO_2_ than site CON in June and July, but in October when temperature was below zero, the CO_2_ fluxes from sites HUM and CON were detected at a similar low level (Table 1).

### Seasonal and diurnal pattern of CO_2_ flux

The CO_2_ flux from all sites showed significant differences among different months (Mann–Whitney test, *P*<0.001), except those from site CON in June and July (Mann–Whitney test, *P* = 0.93).

The CO_2_ flux from all four sites showed significant diurnal changes in June and July (Kruskal Wallis Test, *P*<0.001), except for sites LIT and HUM in July (Kruskal Wallis Test, *P* = 0.05 and *P* = 0.31 respectively). In the winter, the CO_2_ fluxes from sites LIT and HUM varied remarkably (Kruskal Wallis Test, *P*<0.001), while the CO_2_ fluxes from sites INT and CON showed no detectable diurnal difference (Kruskal Wallis Test, *P* = 0.15 and *P* = 0.91 respectively).

### Relationship between environmental factors and ecosystem respiration

**Figure 5:**
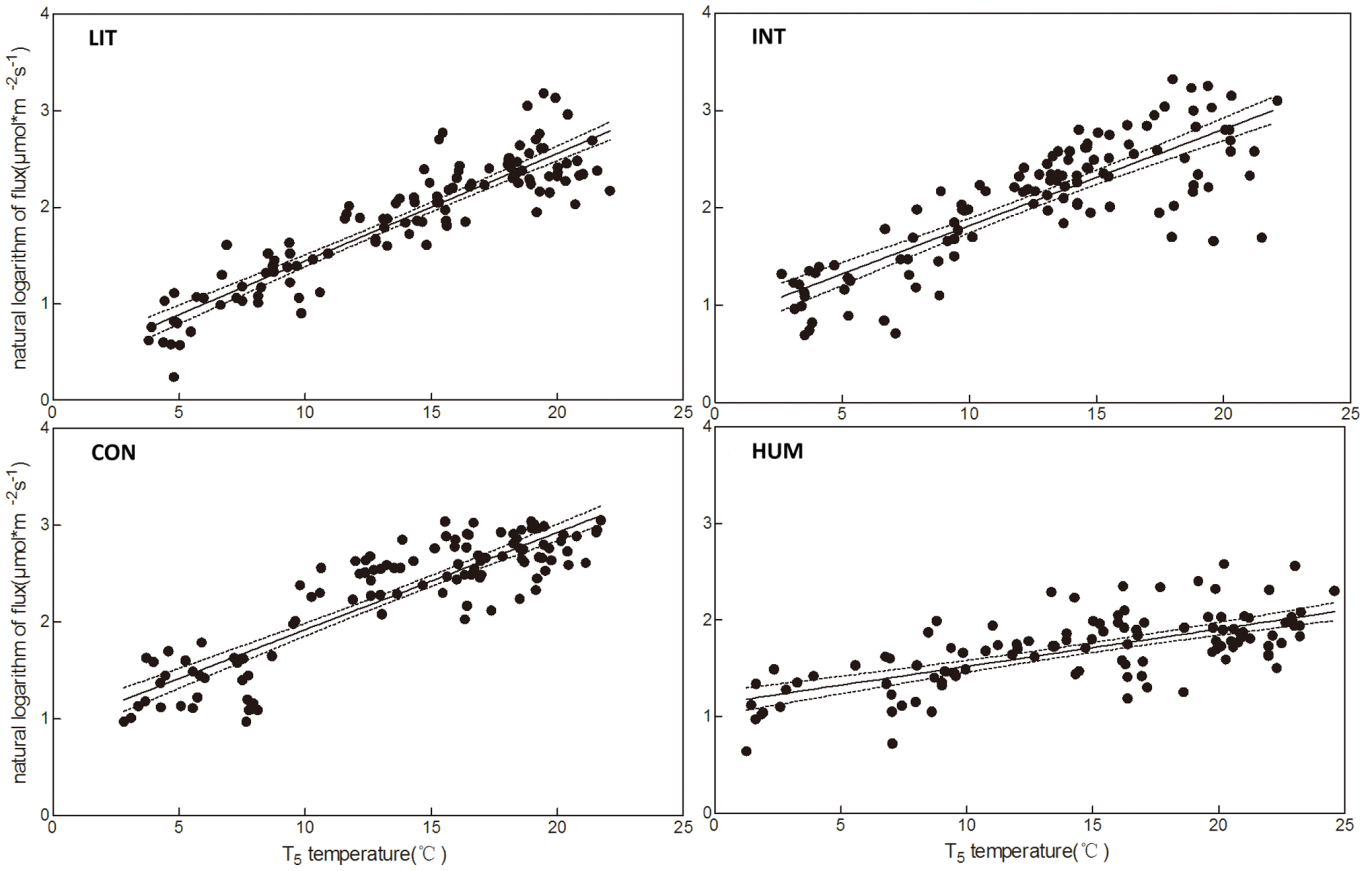
Correlation between ecosystem respiration and T_5_ each groundwater table treatment, as well as for plateau zokor treatment. A linear equation describing the correlation between the natural logarithm of the ecosystem respiration and the soil temperature and the 95% confidence interval are shown: site LIT (*y* = 0.11*x*+0.33, *R* ^2^ = 0.83, *P*<0.001); site INT (*y* = 0.10*x*+0.83, *R* ^2^ = 0.68, *P*<0.001); site CON (*y* = 0.10*x*+0.92, *R* ^2^ = 0.77, *P*<0.001); site HUM (*y* = 0.04*x*+1.13, *R* ^2^ = 0.46, *P*<0.001).

Across all the four study sites, SOC at the 0–10, 10–20 and 20–30 cm layers showed no significant relationship with ecosystem respiration, whereas T_5_ was significantly correlated with ecosystem respiration, and a linear equation could adequately describe the relationship between the natural logarithm of the ecosystem respiration and T_5_ (Fig. 5). According to the parameter of the equation shown in Fig. 5, the respiration of site HUM increased much slower than the other three sites with the increase of T_5_, while site HUM released more CO_2_ at low T_5_ (estimated parameters shown in Table 2). To express the temperature sensitivity, we calculated an apparent Q_10_ [25]. Site HUM had a notably low Q_10_ of 1.47, whilst the other sites were in the range 2.69 to 3.03 (Fig. 5 and Table 2).

**Table 2:** Q_10_ and estimated parameters simulating relationship between flux data and T_5_.

	sites			
	LIT	INT	CON	HUM
Slope (lower 2.5%)	0.10	0.09	0.09	0.03
Slope	0.11	0.10	0.10	0.04
Slope (upper 97.5%)	0.12	0.11	0.11	0.05
Intercept (lower 2.5%)	0.19	0.76	0.65	1.01
Intercept	0.33	0.91	0.83	1.13
Intercept (upper 97.5%)	0.47	1.06	1.00	1.26
Q_10_ (lower 2.5%)	2.76	2.47	2.37	1.36
Q_10_	3.03	2.73	2.69	1.47
Q_10_ (upper 97.5%)	3.34	3.03	3.05	1.59
*P* value	<0.001	<0.001	<0.001	<0.001

With respect to the relationships between respiration and SWC, only the CO_2_ flux from sites INT and CON shown significant correlation with SWC, with the equation, *R* ^2^ and *P* values being [*y* = 3128 exp (−8.993*x*), *R* ^2^ = 0.13, *P*<0.001] and [*y* = 54.93 exp (−3.002*x*), *R* ^2^ = 0.14, *P* = 0.001], respectively, where *y* is the ecosystem respiration (units: umol*m^−2^s^−1^) and *x* is the SWC.

## Discussion

The soil chamber method of evaluating a component of the carbon cycle is broadly applied to explore the impact of small-scale disturbances in the landscape [26], especially the impact of animals on CO_2_ flux [27]. In the Zoige peatlands, where colonies of plateau zokors change the local landscape at a small scale through digging holes, shaping hummocks, and removing plant material, and causing significant changes in the soil biogeochemistry that result in respiration variability. With the soil chamber method, the measurements of respiration collected *in situ* enabled us to describe the impact of plateau zokors on CO_2_ flux.

Many biotic and abiotic factors were closely related to ecosystem respiration, such as plant productivity, temperature and groundwater table [13], [28]. In this study, the descending groundwater table was closely related to the increase of CO_2_ flux from ecosystem respiration (Fig. 4, Table 1), which ascribed to the different SWC among the sites. Higher SWC produced anaerobic environment, prevented aerobic respiration of root biomass and underground soil respiration, and thus sustained more carbon in soil. However, no significant correlation was detected between SWC and ecosystem respiration at site LIT and HUM, which may partly due to the insufficient of replications. At 0–20 cm depth of soil, SOC at site LIT and INT were higher than that at site CON due to the higher groundwater table at site LIT and INT, whilst SOC values were almost the same among the sites at 20–30 cm depth of soil (Fig. 3). SOC was a result of respiratory loss of soil organic matters. Higher respiration rate led to less carbon remained in soil, which is consistent with this study.

We know of only one other study of the effect of burrowing animals on ecosystem respiration [29], which reported the ecosystem respiration was between 4–12 umol*m^−2^s^−1^, and is lower than the values reported in the present study. The previous study found that the ecosystem respiration rate fell with the increase animal population density, since pikas graze on the above-ground vegetation, thus reducing the biomass and also the supply of substrate to the soil microbes. The zokor, in contrast, feeds on roots underground.

Respiration from site HUM was lower and shown less temperature sensitivity, than elsewhere. The low rate of respiration is presumably the result of the general lack of vegetation. The low temperature sensitivity could be explained by assuming that a significant fraction of the respiratory flux detected comes from the animals themselves. It is well-known that the effect of temperature on the respiration rate of mammals tends to work in the opposite direction to the effect of temperature on the respiration rate of plants and microorganisms [30], as warm-blooded organisms need to expend more energy to maintain their body temperature. Quantitative analysis of this effect is not possible yet, as we do not know the population densities of the animals or the specific effect of temperature on their respiration rate. Zokor burrowing activities also reduced respiration rate at site HUM, and transferred deeper soil to surface layer, changed SOC structure (Fig. 3). Low SOC at the surface layer at site HUM might supply less substrate to respiration, and therefore, resulted in low respiration rate.

It is clear from this study that water content, temperature and animal activity jointly determine CO_2_ release from soil. Many other authors focused on the impact of water content, temperature and the quality of the peat on soil respiration [31], [32], while the impact of burrowing animals has rarely been considered. In Zoige Wetland, the density of plateau zokor hummocks is 1350 per hectare [33], accounting for an approximate 3%–5% coverage of the landscape; while in other regions, the density of hummocks is obviously higher [33]. Consequently, when measuring the CO_2_ release of the Zoige peatlands, especially when linking the carbon source to the atmospheric carbon budget, the impact of burrowing animals should be taken into account. In order to precisely clarify zokor's impact on ecosystem respiration, more sites and more replicates maybe represent gradient of zokor density and different vegetation type in future study.

At Zoige, burrowing animals are believed to harm the grazing pasture and compete with cattle for forage [34], [35], while the opposite viewpoint states that burrowing animals are keystone species in local ecosystems [36]. Many studies have focused on the role of burrowing animals in ecosystems, including their impact on biodiversity, water infiltration, and improving soil nutrients [20], most of which are highly relevant to ecosystem respiration. In this study, the SOC in the surface layer of site HUM was less than that of site CON, a pattern which was reversed in the deeper layer (Fig. 3). Although the difference of SOC was not significant here, due to the small size of sampling, it nevertheless suggests that hummocks accelerate C loss in the surface layer, and this may provide better aeration. On the other hand, the absence of vegetation on hummocks may lead to more carbon in deeper soil where microbial activity is lower. Consequently, CO_2_ release from hummocks will exceed that from control sites because of the better aeration in the surface layer, which was more obvious with lower temperature in this study (Fig. 5). When temperature increased, the CO_2_ release from site CON rose more quickly than that from site HUM, inducing much more CO_2_ release from site CON in the growing season (Fig. 4).

The rate of increasing temperature at Zoige has been 0.23°C/10 yr from 1950S [37]. Such rates of warming are likely to result in loss of carbon via the decomposition of peat, leading to a positive feedback from climate warming. The impact of climate warming on the carbon release could to a certain extent be clarified by the Q_10_ value in the present study. An important question to ask is whether Q_10_ values produced from measurements at sites like those described here can be used to predict future respiration rates. Several authors have questioned the applicability of such studies [38], [39]. Ultimately, in the long term, ecosystem respiration is limited by the supply of suitable substrates for respiration, and plant respiration depends on the growth rate of biomass as well as temperature. Thus, as pointed out by Davidson, Janssens & Luo [40], we should realize the limitations of the Q_10_ model in any predictive scheme for future climates.

Ultimately, the function of this burrowing animal on the Qinghai–Tibet Plateau, and its interplay with livestock, other burrowing animals, and soil structure is complex. The present study is preliminary; with much more work to be done in terms of addressing the issue of greenhouse gas emissions. All the variables that control the population density of these animals may change in the future with global warming and the complication of greater human pressure on the resources of the region. How to manage the grazing yaks and the wild burrowing zokors needs further investigation on a larger scale than was possible in the present study.

## References

[1] Gorham E (1991) Northern peatlands - role in the carbon-cycle and probable responses to climatic warming. Ecol Appl 1:182–195. 27755660 10.2307/1941811

[2] Turunen J, Tomppo E, Tolonen K, Reinikainen A (2002) Estimating carbon accumulation rates of undrained mires in Finland-Application to boreal and subarctic regions. Holocene 12:69–80.

[3] Yu ZC, Loisel J, Brosseau DP, Beilman DW, Hunt SJ (2010) Global peatland dynamics since the Last Glacial Maximum. Geophys Res Lett 37.

[4] IPCC (2007) Climate Change 2007: the Physical Science Basis.

[5] Raich JW, Schlesinger WH (1992) The global carbon-dioxide flux in soil respiration and its relationship to vegetation and climate. Tellus Ser B-Chem Phys Meteorol 44:81–99.

[6] Yu Z, Beilman DW, Frolking S, MacDonald GM, Roulet NT, et al (2011) Peatlands and Their Role in the Global Carbon Cycle. Eos, Transactions American Geophysical Union 92:97–98.

[7] Maljanen M, Hytönen J, Martikainen P (2001) Fluxes of N_2_O, CH_4_ and CO_2_ on afforested boreal agricultural soils. Plant Soil 231:113–121.

[8] Beetz S, Liebersbach H, Glatzel S, Jurasinski G, Buczko U, et al (2013) Effects of land use intensity on the full greenhouse gas balance in an Atlantic peat bog. Biogeosciences 10:1067–1082.

[9] Urbanova Z, Picek T, Hajek T, Bufkova I, Tuittila ES (2012) Vegetation and carbon gas dynamics under a changed hydrological regime in central European peatlands. Plant Ecol Divers 5:89–103.

[10] Ives SL, Sullivan PF, Dial R, Berg EE, Welker JM (2013) CO2 exchange along a hydrologic gradient in the Kenai Lowlands, AK: feedback implications of wetland drying and vegetation succession. Ecohydrology 6:38–50.

[11] Fan ZS, McGuire AD, Turetsky MR, Harden JW, Waddington JM, et al (2013) The response of soil organic carbon of a rich fen peatland in interior Alaska to projected climate change. Glob Change Biol 19:604–620. 10.1111/gcb.12041 23504796

[12] Makiranta P, Laiho R, Penttila T, Minkkinen K (2012) The impact of logging residue on soil GHG fluxes in a drained peatland forest. Soil Biol Biochem 48:1–9.

[13] Couwenberg J, Dommain R, Joosten H (2010) Greenhouse gas fluxes from tropical peatlands in south-east Asia. Glob Change Biol 16:1715–1732.

[14] Rochette P, Angers DA, Chantigny MH, Bertrand N, Cote D (2004) Carbon dioxide and nitrous oxide emissions following fall and spring applications of pig slurry to an agricultural soil. Soil Sci Soc Am J 68:1410–1420.

[15] Chadwick D, Pain B (1997) Methane fluxes following slurry applications to grassland soils: laboratory experiments. Agriculture, ecosystems & environment 63:51–60.

[16] Rodhe L, Pell M, Yamulki S (2006) Nitrous oxide, methane and ammonia emissions following slurry spreading on grassland. Soil Use Manage 22:229–237.

[17] Rochette P, Angers DA, Chantigny MH, Gagnon B, Bertrand N (2008) N_2_O fluxes in soils of contrasting textures fertilized with liquid and solid dairy cattle manures. Can J Soil Sci 88:175–187.

[18] Zhang YM, Zhang ZB, Liu JK (2003) Burrowing rodents as ecosystem engineers: the ecology and management of plateau zokors Myospalax fontanierii in alpine meadow ecosystems on the Tibetan Plateau. Mammal Rev 33:284–294.

[19] Zhang YM, Liu J (2003) Effects of plateau zokors (*Myospalax Fontanierii*) on plant community and soil in an alpine meadow. J Mammal 84:644–651.

[20] Davidson AD, Detling JK, Brown JH (2012) Ecological roles and conservation challenges of social, burrowing, herbivorous mammals in the world's grasslands. Front Ecol Environ 10:477–486.

[21] Hogan BW (2010) The plateau pika: A keystone engineer on the Tibetan Plateau. Tempe, Arizona: Arizona State University.

[22] Chen H, Wu N, Yao SP, Gao YH, Zhu D, et al (2009) High methane emissions from a littoral zone on the Qinghai-Tibetan Plateau. Atmos Environ 43:4995–5000.

[23] Semenov MV, Kravchenko IK, Semenov VM, Kuznetsova TV, Dulov LE, et al (2010) Carbon Dioxide, Methane, and Nitrous Oxide Fluxes in Soil Catena Across the Right Bank of the Oka River (Moscow Oblast). Eurasian Soil Sci 43:541–549.

[24] Inc S (2009) PASW Statistics 17.0. SPSS Inc., Chicago, Illinois, US.

[25] Lloyd J, Taylor J (1994) On the temperature dependence of soil respiration. Funct Ecol 8:315–323.

[26] Zhang L, Chen Y, Zhao R, Li W (2012) Soil carbon dioxide flux from shelterbelts in farmland in temperate arid region, northwest China. Eur J Soil Biol 48:24–31.

[27] Chen WW, Wolf B, Bruggemann N, Butterbach-Bahl K, Zheng XH (2011) Annual emissions of greenhouse gases from sheepfolds in Inner Mongolia. Plant Soil 340:291–301.

[28] Jagermeyr J, Gerten D, Lucht W, Hostert P, Migliavacca M, et al (2014) A high-resolution approach to estimating ecosystem respiration at continental scales using operational satellite data. Glob Change Biol 20:1191–1210. 10.1111/gcb.12443 24259306

[29] Liu YS, Fan JW, Harris W, Shao QQ, Zhou YC, et al (2013) Effects of plateau pika (*Ochotona curzoniae*) on net ecosystem carbon exchange of grassland in the Three Rivers Headwaters region, Qinghai-Tibet, China. Plant Soil 366:491–504.

[30] Schmidt-Nielsen K (1997) Animal physiology: adaptation and environment. Cambridge University Press.

[31] Leifeld J, Steffens M, Galego-Sala A (2012) Sensitivity of peatland carbon loss to organic matter quality. Geophys Res Lett 39.

[32] Juszczak R, Humphreys E, Acosta M, Michalak-Galczewska M, Kayzer D, et al (2013) Ecosystem respiration in a heterogeneous temperate peatland and its sensitivity to peat temperature and water table depth. Plant Soil 366:505–520.

[33] Chen L, Liao WB, Yang ZS, Zhang Y, He Y, et al (2010) The space distribution of plateau zokor hummocks. Journal of China West Normal University (Natural Sciences) 31:122–125 (in Chinese).

[34] Fan N, Zhou W, Wei W, Wang Q, Jiang Y (1999) Rodent pest management in the Qinghai-Tibet alpine meadow ecosystem. In:G. RSingleton, L. AHinds, LLeirs and ZZhangeditors. Ecologically-based management of rodent pests. Canberra: Australian Centre for International Agricultural Research. pp.285–304.

[35] Limbach WE, Davis JB, Bao T, Shi D, Wang C (2000) The introduction of sustainable development practices of the Qinghai Livestock Development Project. In:D Zheng editor. Formation and Evolution, Environmental Changes and Sustainable Development on the Tibetan Plateau. Beijing: Academy Press. pp.509–522.

[36] Delibes-Mateos M, Smith AT, Slobodchikoff CN, Swenson JE (2011) The paradox of keystone species persecuted as pests: A call for the conservation of abundant small mammals in their native range. Biol Conserv 144:1335–1346.

[37] Luo Q, Peng GZ (2008) The impact of climate change around Zoige on wetland environment. Plateau and Mountain Meteorology Research: 44–48. (in Chinese)

[38] Giardina CP, Ryan MG (2000) Evidence that decomposition rates of organic carbon in mineral soil do not vary with temperature. Nature 404:858–861. 10786789 10.1038/35009076

[39] Grace J, Rayment M (2000) Respiration in the balance. Nature 404:819–820. 10786772 10.1038/35009170

[40] Davidson EA, Janssens IA, Luo YQ (2006) On the variability of respiration in terrestrial ecosystems: moving beyond Q(10). Glob Change Biol 12:154–164.

